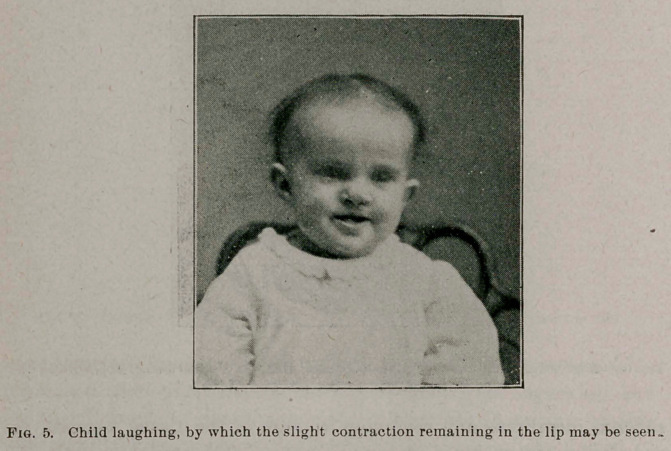# A Case of Unusual Defective Development in an Infant

**Published:** 1898-01

**Authors:** John O. Roe

**Affiliations:** Rochester, N. Y.


### BUFFALO MEDICAL JOURNAL.

VOL. XXXVII. JANUARY, 1898. No. 6.

### Original Communications.

### A CASE OF UNUSUAL DEFECTIVE DEVELOPMENT IN AN INFANT.

CONSISTING OF COMPOUND COMPLICATED DOUBLE HARELIP, AND BILATERAL MAXILLARY FISSURE, WITH ATTACHMENT OF THE INTERMAXILLARY BONE TO THE END OF THE NOSE: CORRECTION OF THIS DEFORMITY BY PLASTIC OPERATIONS.[1]

BY JOHN O. ROE, M. D., Rochester, N. Y.

THE following case of unusual defective facial development, which I am about to detail to you, has been of so much interest to me, both on account of the extent of the deformity and of the excellent results attending the operations for its correction, that I thought it worthy of your consideration :

By the advice of Dr. Henry S. Benham, of Honeoye Falls, N. Y., a child, 5 weeks old, was brought to me from West Bloomfield, N. Y., January 6, 1897, by its parents, who wished to ascertain if anything could be done to correct a very extensive facial deformity with which the child had been born. On examination, the child was found to have a double harelip, a bilateral maxillary fissure, and a wide cleft through both hard and soft palate. This space in the jaw and upper lip was so wide that upon inspecting the mouth, the nasal passages and turbinated bodies, together with the nasal septum, were plainly to be seen ; the latter hanging down between the turbinated bodies like a center-board, but deflected to the left. To the anterior portion of the septum and the end of the nose was attached the intermaxillary bone, or osseous tubercle, which, together with the integumentary tissue, should have formed the central portion of the jaw and lip.

1. Read at the thirtieth annual meeting of the Medical Association of Central New York, at Buffalo, October 19, 1897.

The parents of the child were both perfectly well and their other child, 2 years old, was also perfectly normal. On questioning them as to the probable cause of this deformity, they stated that they could learn of no case of deformity or malformation that had ever occurred in either family, but believed this one to be due to maternal impression.

The mother said that when she was about two and a half months advanced in pregnancy her little girl, then about one year old, was laid in her arms just as it was going into a spasm. The child was gasping for breath, and working its mouth, and seemed to be dying. The strain from this excitement was so severe that she did not get over it for a long time. About two months after this occurrence, she was very much frightened by her little girl getting a pin in its throat, which she, being

FIG. 1. Child as it appeared when five weeks old, before any operative interference.

alone with the child, was obliged to remove with her finger. After this occurrence the mother felt sure that her child would be marked and her fears were fully realised.

The child, when brought to me, presented a very repulsive appearance, and the deformity seemed a very formidable one, so far as its correction was concerned. Owing to the large opening in the central portion of the jaw and lip, the child was not only unable to nurse, but was unable even to take food from a spoon, and the only satisfactory way of feeding it was to drop the milk into its mouth with a dropper ; and in this manner it was fed from the hour of its birth until the restoration of the upper jaw and lip. Notwithstanding this disability, the child enjoyed good health to an unusual degree. It has suffered from none of the common infantile ailments, and up to this time, the mother states, it has not seen a sick day.

Owing to the excellent condition of the child, I advised that the operations necessary for the correction of this deformity be undertaken at once. Accordingly, the following week the child was brought to my office and on January 9th I performed the first operation. This operation consisted in bringing down into place the intermaxillary portion of the jaw, which was attached to the end of the nose. On attempting to force this intermaxillary bone downward and backward into place, the lower border of the cartilaginous septum necessarily wrinkled more or less and bent to one side.

It was, therefore, apparent that the plan generally proposed by surgeons, of forcibly breaking down this portion sufficiently to overcome its elasticity, was not only unadvisable but impractica-

Fig. 2. Representing the cleft through the hard and soft palate, showing the attachment o1 the intermaxillary bone to the end of the septum.

ble. To obviate this difficulty, I took out a V-shaped portion of the lower border of the cartilaginous part of the septum, according to the plan proposed by Blandin, at the same time incising beneath the soft parts the upper border, just anterior to the incision already made. The bone was then readily brought down into place on a line with the superior maxilla, and sutured to the left side of the jaw with silver wire, the two surfaces being freshened and shaved off flat and smooth so that they co-aptated perfectly throughout.

As the intermaxillary bone was only large enough to fill say about one-half of the gap in the upper jaw and, as no other osseous tissue was available for filling in the remaining portion, the thought occurred to me that it might readily be closed by slowly forcing the parts together by means of elastic pressure, applied on the outside of the jaw. This plan was especially feasible for the reason that the child's face was naturally quite wide and a slight narrowing of the upper jaw would not make the face appear contracted or distorted. This plan was carried out with complete success, and by the 1st of March this gap in the jaw was completely closed.

When the surfaces on this side had come firmly together, they were scarified and shaved off smoothly so that they would co-aptate perfectly, as was done on the other side, and securely sutured together with silver wire.

These silver wire sutures caused no ulceration or irritation, thorough antiseptic precautions having been instituted when they

FIG. 3. Representing closure of maxillary fissure and the maxillary bone *in situ*.

were inserted; and they were allowed to remain for a considerable time, until the operations for the restoration of the upper lip had been performed, to guard against any possible loosening of this intermaxillary portion, and until it had become solidly united to the superior maxilla.

In the early part of April, the operations for the restoration of the upper lip were undertaken. As before stated, this distorted portion was covered with integument which should have formed the central portion of the lip, and which proved exceedingly serviceable for filling in the deficient floor of the nostrils and in forming the vermilion border for the lip. The floor of the nostrils was made first. This was done by uniting two flaps, one taken from the upper half of the maxillary nodule and the other from the upper and inner surface of the cleft.

When the union of these two flaps had taken place, the slight comb, or ridge, formed along their line of union was pared down so as to leave the floor of the nostrils perfectly smooth. In this manner the floors of both nostrils were formed, so that at the present time one would not suspect that they had not been normal from the first.

The cartilaginous portion of the septum, where the osseous tubercle had been united, was still very much too thick, and the skin of the column forming the anterior portion of the septum was too short, so that it drew the end of the nose downward, flattening it somewhat. This thickening was removed by lifting the skin and

FIG. 4. Representing child at ten months of age, after the completion of the work in the restoration of the jaw and lip.

mucous membrane and removing enough of the tissue beneath to reduce the septum to its normal thinness. The two sides were not operated upon, however, at the same time; one being allowed to heal before the other was disturbed. The anterior column was then lengthened sufficiently by making an oblique incision through it and sliding the flaps.

When the nostrils were completed, the lower border of the lip was formed. This was done according to Malgaigne's method by making flaps; two from the lower half of the intermaxillary nodule, one from either side, and one from the lip on each side. These were turned downward so that the raw surfaces coaptated and were sutured together carefully so that the vermilion border of the lip was accurately formed, but made slightly more prominent at the point of union of the two flaps, to allow for contraction on healing.

A central aperture on the right side still remained. This was closed by scarifying and freshening the edges and suturing them together, the coaptation of the edges being aided by a compress, similar to Hainsley's compressor, on the outside of the face to prevent traction on the sutures.

The upper lip at first was very much shorter than the lower, but, as the child develops, this is gradually lengthening, so that at the present time but very little contraction of the lip is apparent to the casual observer, (as shown by the photograph of the child, taken last week, Fig. 5) and the scar is quite rapidly fading away.

FIG. 5. Child laughing, by which the slight contraction remaining in the lip may be seen.

Later on, should the upper jaw be found to be too narrow, so that mastication is interfered with, a portion of the centre of the lower jaw can be resected sufficiently to make the two jaws sustain their normal relations to each other.

The eruptation of the teeth has gone on quite normally and one well-developed tooth has appeared in the intermaxillary portion. The child has been for some time able to take the nipple of the bottle, and is fed in the usual manner, with no regurgitation of the food through the nose, and there have been no untoward complications.

All these operations which the child has undergone have been performed under complete anesthesia from chloroform, but notwithstanding this, the child has suffered no ill effects from its use whatever, and has not been disturbed by nausea following its administration ; also, the loss of blood has been so slight that the child has not been weakened by it in the least. The child has been so unusually good, and its appearance has improved to such a remarkable degree, that she has been dubbed, "The Belle of Bloomfield."

The only operation remaining yet to be done is the closure of the cleft in the palate. This has been deferred until the other portion of the work was completed and also to give time for the development of the tissues, for, as we know, many very wide clefts become very much narrowed during development.

It is important, however, that the closure be done before the child begins to talk, so that the child will not acquire an abnormal or perverted method of speaking.

The points in this case which are especially worthy of notice are :

1. The very wide double cleft through the upper lip, the jaw and the hard and soft palate, and the free exposure of the nasal chamber.

2. The size and amount of projection of the intermaxillary bone, and its attachment to the end of the nose.

3. The complete success attending the loosening and bringing down of this intermaxillary portion and its firm union, on both sides, to the maxilla.

4. The facility with which the aperture left in the jaw (which this intermaxillary bone was too small to fill) was closed by elastic tension applied on the outer side of the jaw.

5. The success which has attended the operations for constructing the floor of the anterior portion of the nostrils and filling in the gap in the lip, giving it quite a normal appearance.

There are two points in this subject on which surgeons differ in their opinions. One is the question of removing or leaving the premaxillary bone in cases of compound complicated harelip ; the other is the question as to the age at which the operation had best be performed.

In regard to the former question, the very best authorities differ widely. Many surgeons are of the opinion that when the premaxillary bone projects too much, it should be removed, while others believe it is of the highest importance that this portion of the bone, if possible, be preserved.

The reasons advanced by surgeons, who advocate its removal, are :

1. That pressing the premaxillary bone back into place is difficult and unsatisfactory ; and if pressed back it rarely unites to the maxillæ with bone and, in that case, will interfere with the closure of the alveolar arch and palate fissure.

2. That the teeth which it should contain (the central incisors) cannot be relied upon to come through in such a manner as to be serviceable, and that a plate made by a dentist will answer the purpose quite as well.

Other surgeons urge the preservation of the premaxillary bone for the following reasons :

1. That if the bone be removed, there must be a permanent gap through the hard palate and in consequence a flattening and malposition of the upper lip, from having lost its bony support.

2. That this flattening of the jaw will make the lip short and tense and the patient very much "underhung," an unsightly deformity.

3. That the presence of this bone is needed to preserve the normal width and arch of the jaw and best fits it for carrying artificial teeth, if such are needed, because of the unsatisfactory eruption of the natural teeth.

The advisability of preserving the premaxillary bone, it would seem, cannot be questioned, and it is in very exceptional cases only that its removal is required. Some surgeons regard its removal as but little short of an unjustifiable mutilation.

The second question, "as to the age at which the operation for harelip had best be performed," has been a subject of discussion from the earliest surgical time, and there has been no age from one day to five years, which has not been advocated by some surgeon as the most desirable time for the operation.

There are two points, however, upon which surgeons are pretty generally agreed ; these are that the condition of the lesion and the health of the patient should govern the time for operating. Some surgeons advocate that the operation be performed as quickly as possible after birth, while others claim that the best time is from the fifth to the tenth week.

When the child is vigorous, well-nourished and well-cared for, the early operation is preferable ; but the principal danger in operating on the newly born infant is from hemorrhage, for the reason that very young children suffer severely from the loss of blood and, in some cases, the loss of a very small amount of blood proves fatal.

It is advisable, however, in all cases, to operate as early as is consistent with safety to the child, in order to interfere as little as possible with the eruption of the teeth; for, after this has begun, all operative interference should be deferred until the first dentation is completed.

28 NORTH CLINTON STREET.

## Figures and Tables

**Fig. 1. f1:**
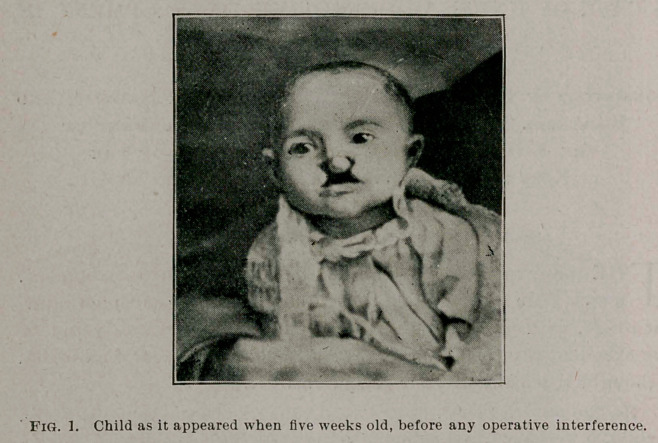


**Fig. 2. f2:**
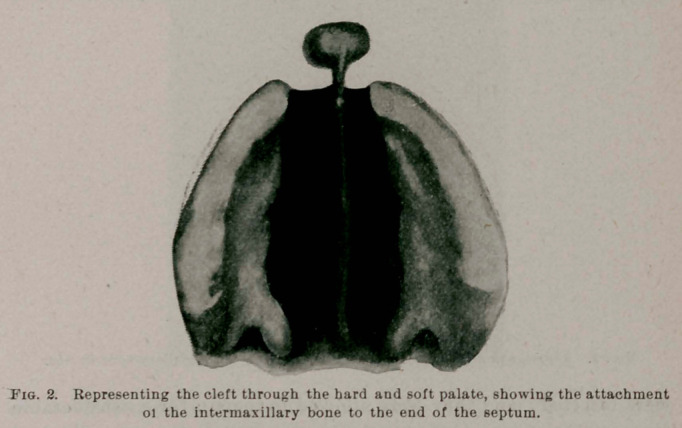


**Fig. 3. f3:**
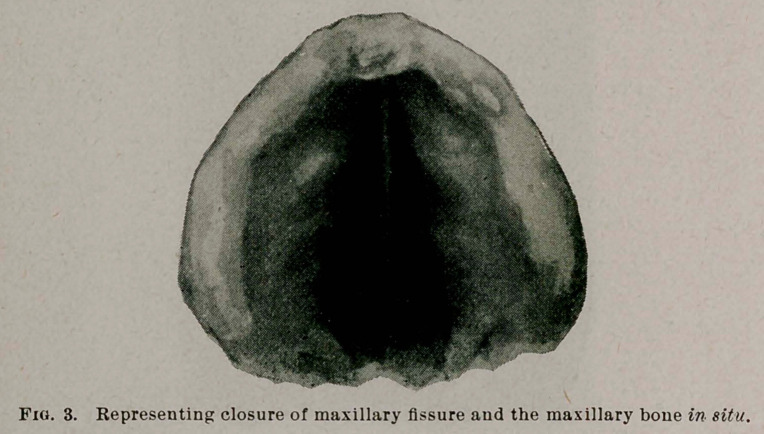


**Fig. 4. f4:**
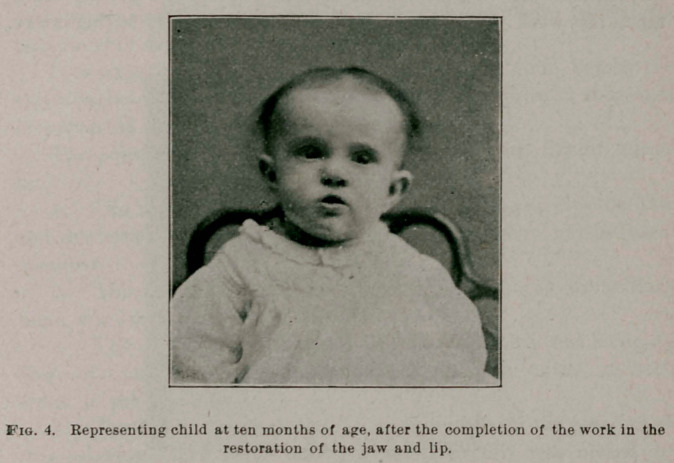


**Fig. 5. f5:**